# Functionalized Gold Nanoparticles for Facile Pattern-Controlled Surface Coatings

**DOI:** 10.3390/biomimetics9030146

**Published:** 2024-02-28

**Authors:** Jue Wang, Junfeng Liang

**Affiliations:** Department of Chemistry and Chemical Biology, Stevens Institute of Technology, Hoboken, NJ 07030, USA; jwang121@stevens.edu

**Keywords:** patterned coating, gold nanoparticles, dopamine, adhesive nanoparticles

## Abstract

Gold nanoparticles (AuNPs) have been widely investigated as surface modifiers; nevertheless, most methods still require the pretreatment of surfaces and several steps to control coating efficiency and patterns for improved functionality. We developed functionalized AuNPs through borate-protected dopamine (B-AuNPs). The simple activation of B-AuNPs with a strong acid to remove the protected borate groups produces adhesive dopamine AuNPs (D-AuNPs). D-AuNP-coated surfaces with varied but controlled features and properties such as coating density and surface pattern were achieved using D-AuNPs with a precisely controlled dopamine density and coating conditions. Such adhesive and easily manipulated AuNPs provide a facile and time-saving technology to achieve sophisticated surface coatings using AuNPs.

## 1. Introduction

Nanoparticles possess different properties compared to bulk materials, making them applicable in various areas. Among the abundant types of nanoparticles, noble metals, especially gold nanoparticles (AuNPs), have been intensively investigated, as they present distinct absorbance and fluorescence from visible to the near-infrared (NIR) region [1,2], potential for several types of functionalization [3], easily manipulated synthesis [4,5], and biocompatibility [6]. These properties endow AuNPs with great potential for numerous applications such as sensing, cancer therapy, antimicrobials, enzymatic mimics, and surface modifications [7,8,9,10,11,12]. AuNP-modified surfaces play important roles in various areas. For example, AuNP-coated surfaces are widely used in medical devices to prevent potential infections and biofilm generation [13,14]. Furthermore, they are used to obtain better optical properties and surface conductance [15,16,17]. Thus, AuNPs provide an excellent platform for surface functionalization.

To obtain modified surfaces for AuNP immobilization, the treatment of AuNPs and surfaces proceeds normally. Physical methods such as metal evaporators and electron beam evaporation are employed to apply gold on substrates [18,19]. The pretreatment of surfaces includes amine- or sulfur-functionalized substrates, a sputtered gold layer, and then blocking by hexane-1-thiol, polymer graft, or an adhesive layer on the surface [19,20,21,22]. Alternatively, reducing surfaces in situ to reduce gold ions to AuNPs can also be performed [23]. Post-treatment includes using Nafion to hold AuNPs after deposition or simply heating the surface for stronger immobilization [24,25]. Moreover, using linker compounds such as 1,9-nonanedithiol to continuously adhere AuNPs on surfaces has also been reported [26].

Surface-modified AuNPs have also been intensively investigated to expand their usage and to overcome limitations. Functionalized AuNPs include different sizes and ligands contributing to various areas, especially sensing and biomedical applications [27,28]. Small molecule [29], antibody, and nucleic acid modifications provide novel detection and specific therapeutic possibilities [30,31]. Numerous methods were developed for AuNP surface engineering such as using sulfur-containing ligands, phosphine, amine, polymers, or other adhesive ligands [32]. Other methods, such as mechanical extrusion, N-hydroxysuccinimide-activated ester, and electrostatic adhesion, were also reported in [33,34]. Among these strategies, sulfur–Au bonds are commonly selected [35,36,37].

**Scheme 1:**
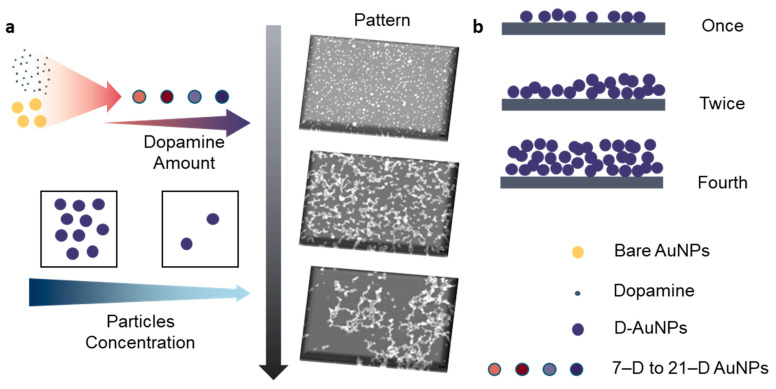
Pattern-controlled coating mechanism. (**a**) Patterned coating manipulated through the dopamine amount on the AuNP surface and AuNP concentration in the coating system. (**b**) Sideview scheme of different aggregation levels.

In this research, dopamine was selected as a surface anchor for AuNPs, inspired by mussel byssus [38,39]. The catechol group of dopamine can involve many reactions and is the essential component of adhesivity [40,41]. Carbon disulfide (CS2) was linked to the primary amine of dopamine, introducing rapid and robust interactions with AuNP surfaces [42,43]. As the catechol group of dopamine was protected by forming dithiocarbamate (B-DDTC), this resulted in colloidal-stable self-adhesive AuNPs. Such borate-protected AuNPs (B-AuNPs) can be activated by concentrated hydrochloric acid (HCl) to become adhesive AuNPs (D-AuNPs) for manageable surface coatings. The coating patterns using such adhesive AuNPs can be manipulated and tuned by controlling dopamine density-varying AuNP concentrations (Scheme 1).

This new approach provides a faster and simpler method for nanoparticle immobilization, reducing the processing time and complexity, and has the potential for various applications that require diverse AuNP surface coating.

## 2. Materials and Methods

### 2.1. B-AuNP Synthesis and Characterization

AuNPs were synthesized by employing a previously developed method from gold (III) chloride (HAuCl_4_), using sodium borohydride (NaBH_4_) as the reducing agent [43]. First, 1 mM of HAuCl_4_ in 10 mL Milli Q water was prepared. Then, 0.627 mg NaBH_4_ was weighed and made into a 600 μL aqueous solution with Milli Q water. The fresh NaBH_4_ solution was added into the HAuCl_4_ solution dropwise under vigorous stirring and reacted for at least 2 min. The light-yellow solution then turned into a wine-red color bare AuNPs solution, and the bare AuNPs were obtained. Then, dopamine monohydrochloride was dissolved in 1.5 mL of a pH 10 borate buffer (0.1 M Na_2_B_4_O_7_·10H_2_O, 50 mM NaOH, 0.1 M NaCl), and the dopamine amount was adjusted as desired. The reaction mixture was then sonicated for 5 min to get borax protected dopamine. After that, 10 μL CS_2_ (for 26.4 mg dopamine) was added and sonicated for another 10 min until cloudy precipitation acquired. The 1.5 mL of borate-protected dopamine dithiocarbamate (B-DDTC) solution was added to a previously prepared bare AuNP solution, and the mixture was stirred at room temperature for more than 15 min to obtain B-AuNPs. Basic physical characterization was achieved via UV–vis spectroscopy (BioTek, Winooski, VT, USA, μQuant microplate spectrophotometer and Agilent 8453 spectrophotometer) and dynamic light scattering (DLS, Zetasizer Nano ZS, Malvern Panalytical, Malvern, UK) for hydrodynamic diameter and zeta potential. As prepared B-AuNPs were diluted in ultra pure water 10 times before proceeding DLS in order to avoid error caused by solution color and absorbance. B-AuNPs were harvested from reaction mixture by solvent exchange by adding same amount of pure ethanol followed by centrifugation at 3000 rpm for 20 min. Resulted supernatant were collected to monitor DDTC binding efficiency, and B-AuNPs were resuspended in desired solvents such as 70% ethanol/water solution, water, etc.

### 2.2. Adhesive AuNP Activation and Controllable Surface Coating

As-prepared borax-protected dopamine dithiocarbamate functionalized gold nanoparticles (B-AuNPs) were activated prior to surface coating by washing with Milli-Q water adjusted to pH 3 using concentrated HCl. This removes the borax protection from the dopamine ligands on the nanoparticle surface. The B-AuNPs were gently shaken in acidic solution for 30 min at room temperature followed by centrifugation at 3000 rpm for 20 min, DDTC-AuNPs were washed again using pH 3 water and resuspended in a desired solvent such as 70% ethanol/water solution, water, etc. Coating substrates include silicon wafer, cover slides and well-plates were immersed into D-AuNPs in a 70% ethanol/water solution for various time periods and in different concentration to control coating patterns, then rinsed with a 70% ethanol/water solution, and air-dried. Scanning electron microscopy (SEM, Zeiss Auriga small dual-beam FIB-SEM, Oberkochen, Germany) images of DDTC-AuNP coated silicon wafer samples were obtained at an accelerating voltage of 3 kV and a working distance at 3 mm. Gold sputter coating was not necessary to image the nanoparticles since they are electron dense. Surface morphology and height measurements were conducted using an atomic force microscope (AFM, Bruker’s BioScope Resolve BioAFM, Billerica, MA, USA). AFM measurements were carried out with 10 × 10 and 1 × 1 μm scan areas. AFM images were taken in the tapping mode using a standard force modulation AFM probe (HQ:NSC19/Al BS) with D-AuNPs coated silicon wafers.

### 2.3. Image Process and Statistical Analysis

The SEM images of the DDTC-AuNPs coatings were processed using ImageJ Fiji (version 2.15.0) software to extract nanoparticle size and density across the substrate surfaces. The AFM images acquired of DDTC-AuNP coated substrates were analyzed using NanoScope Analysis software (version 2.0) to process the surface morphological data and quantify parameters such as surface roughness. The hydrodynamic size distribution and zeta potential data collected from DLS measurements were processed with the instrument’s software (Zetasizer software version 8.02) to obtain statistical size information including the size distribution and polydispersity index (PDI) as well as zeta potential values. All data gathered, including SEM, AFM, and DLS, were plotted using OriginPro 2021 (version 9.8.0.200) and analyzed with built-in statistical analysis toolkit.

## 3. Results and Discussion

### 3.1. Synthesis of Dopamine-Grafted AuNPs with Varied Dopamine Densities

**Figure 1:**
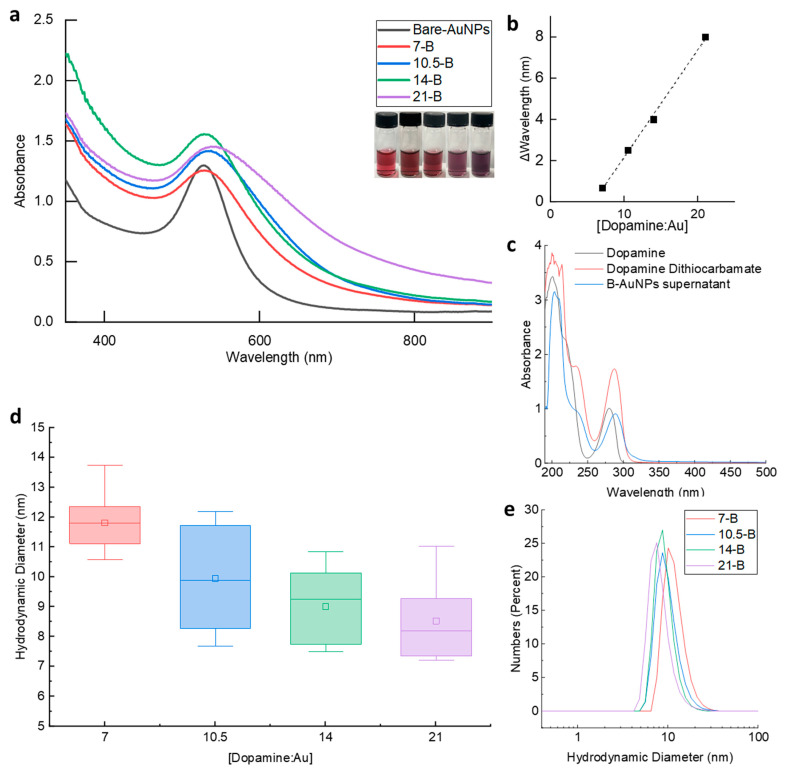
Dopamine-modified AuNP characterization using a UV–vis spectrum and DLS. (**a**) UV–vis spectrum of different B-AuNPs and bare AuNPs in the original synthesis solution; picture of B-AuNPs in the original reaction solutions, from left to right: bare AuNPs, 7–B, 10.5–B, 14–B, 21–B AuNPs. (**b**) Linear fit of B-AuNP absorbance peak position shifted wavelength compared to its original bare AuNPs (R^2^ = 0.99). (**c**) UV spectrum reference of dopamine, dopamine dithiocarbamate (DDTC), and supernatant after 14-B AuNP centrifugation. (**d**) B-AuNPs’ hydrodynamic diameter in water for different [dopamine: Au] molar ratio. (**e**) Typical hydrodynamic diameter (nm) number distribution curve of B-AuNPs.

Grafting dopamine to the surfaces of AuNPs was accomplished through a dithiocarbamate-based AuNP functionalization approach using carbon disulfide (CS_2_) of good stability and efficiency [42]. To avoid potential interference and auto-oxidation, the catechol group of dopamine was protected with borate prior to the reaction. The resulting AuNPs were named as B-AuNPs. Four types of B-AuNPs with varied dopamine-to-Au molar ratios were synthesized and comprehensively investigated. The dopamine amounts used here were 13.2, 19.8, 26.4, and 39.6 mg, given 0.07, 0.105, 0.14, and 0.21 mmol. This produced different dopamine to Au precursor molarity ratios a total of 7, 10.5, 14, and 21 times in original reaction mixture. Thus, the corresponding B-AuNPs acquired were labeled as 7-B, 10.5-B, 14-B, and 21-B AuNPs, although the final ratio would vary. The absorption spectroscopy and dynamic light scattering (DLS) were employed to confirm the successful dopamine grafting on AuNPs. The UV–vis spectrum detected the surface plasmon resonance (SPR) of AuNPs, which reflected their surface properties, sizes, shapes, and aggregations [44]. The attachment of dopamine to the AuNP surfaces led to a change in the maximal peak position and shape in UV–vis spectrum. The introduction of dopamine made the peak position shift further to the red-light area (Figure 1a), from 527 nm for the bare AuNPs to 540 nm for the 21-B AuNPs. The shifted peak wavelengths were linear with the [dopamine: Au] molar ratio. The absorption peak of the bare AuNPs was observed at 527 nm, while the peak of the B-AuNPs was located 1–8 nm further from the bare AuNP peak on average. The peak for 21-B AuNPs were observed at 540 nm. The increased part compared to bare AuNPs was linear to the concentration ratio of dopamine and Au, with an R^2^ = 0.99 (Figure 1b). The B-AuNPs in the original solutions showed a clear color change from red to purple, providing a visual indication of a successful modification (Figure 1a). To further confirm the production of dopamine dithiocarbamate (DDTC) and its binding efficiency to AuNP surfaces, representative peaks of dopamine and DDTC were monitored by UV-range spectroscopy (Figure 1c). Free dopamine showed its typical peak at 280 nm while DDTC shifted this peak close to 287–290 nm. The supernatant after the B-AuNPs were harvested by centrifugation also detected a DDTC peak with about half intensity. Considering the same dilution level was used during the UV spectroscopy, the ligation of DDTC to AuNPs was successful but the efficiency can be improved. In case of solvent affect optical spectrum accuracy, the UV-vis spectrums of dopamine and DDTC were obtained both in water and 50% ethanol/water solution to obtain comparable data especially for supernatant of B-AuNPs washed with ethanol.

The hydrodynamic diameters (*d_H_*) of the B-AuNPs were also tested using DLS, which accurately reflected the ligand conjugation status on AuNPs, especially the spherical particles [45]. Unlike the UV–vis spectrum results, the hydrodynamic diameter of B-AuNPs kept decreasing along with incremental dopamine amount, indicating increased diffusion coefficients in aqueous condition (Figure 1d). This is in agreement with the fact that borate has extremely hydrophilic molecules and can be easily solvated by water molecules in solution, thus, endowing the B-AuNPs with great stability and solubility. Such intensive interactions with water molecules result in elevated diffusion speed and reduced *d_H_*, because of the inverse proportional relationship between *d_H_* and diffusion coefficients. With varied surface modifier amounts, the B-AuNPs presented close but statistically different *d_H_* with unchanged core sizes (Figure 1d). The number distribution curve represented typical B-AuNPs *d_H_* range; such sharp peaks suggest good size unity (Figure 1e).

**Figure 2:**
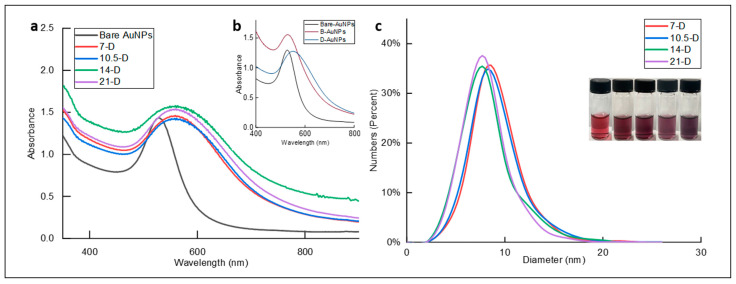
Characterization of D-AuNPs using a UV–vis spectrum and SEM. (**a**) UV–vis spectrum of different D-AuNPs and bare AuNPs in water. (**b**) Typical absorbance spectrum of B-AuNPs, D-AuNPs, and bare AuNPs. (**c**) Diameter distribution of different D-AuNPs measured from the SEM images; picture of D-AuNPs in a 70% ethanol/water solution, from left to right: bare AuNPs, 7–D, 10.5–D, 14–D, 21–D AuNPs.

Following the synthesis of B-AuNPs, pH 3 water adjusted with concentrated hydrochloric acid (HCl) was used to remove borate protection and to release the active catechol group of dopamine without affecting DDTC stability and conjugation on AuNPs, leading to bio-adhesive AuNPs (D-AuNPs) [42]. Following the naming of the B-AuNPs, D-AuNPs with different dopamine amounts were named according to their precursors (7–D, 10.5–D, 14–D, and 21–D AuNPs). D-AuNPs exhibited good dispersion stability if resuspended in 100% ethanol or 70% ethanol/water solution. However, unlike B-AuNPs, D-AuNPs in water showed significantly shifted and wide peaks with a long wavelength (Figure 2b), evidenced by the formation of small but evenly distributed AuNP clusters. The D-AuNPs of different dopamine densities were easily distinguished from bare AuNPs and each other in the 70% ethanol/water solution according to solution colors (Figure 2c). Since the D-AuNPs were reactive, they did not exhibit great dispersity for DLS measurement, and the core size was acquired from SEM images. The D-AuNP size was mostly distributed in the 6–10 nm range, and there was no significant difference between each group at *p* < 0.05 (Figure 2c). In addition to the DLS result of B-AuNPs, DDTC modification was successfully achieved on AuNP surfaces on various levels without affecting core structures.

### 3.2. Surface Coatings Using D-AuNPs

**Figure 3:**
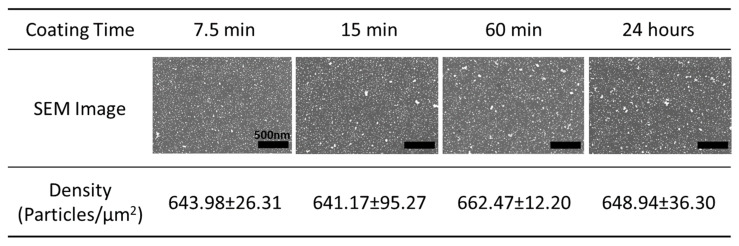
D-AuNP coating in a 70% ethanol/water solution. SEM of 0.5 mM 14–D in a 70% ethanol-solution-coated surface for 7.5, 15, 60 min, and 24 h. (Scale bar: 500 nm).

Unlike B-AuNPs, D-AuNPs demonstrated significant adhesivity to various surfaces. For better controlled surface coating, 70% ethanol/water solutions instead of pure water were used to reduce the reactivity of D-AuNPs. The substrates tested were mainly silicon wafers, although glass and plastic substrates were also effectively coated by D-AuNPs. The results indicate that D-AuNPs could achieve rapid coating within 7.5 min. Specifically, a high concentration (0.5 mM) of D-AuNPs exhibited a rapid coating rate, and no significant difference in the numbers of nanoparticles counted from the SEM images was observed between short and long coating times (Figure 3). Moreover, a shorter coating time resulted in a neater surface with fewer aggregated pieces, which is the bigger bright dot in the pictures. To quantify the D-AuNP surface coverage, nanoparticle numbers in a particular SEM area were counted using software ImageJ Fiji (version, 2.15.0). From 7.5 min to 24 h, the average density of D-AuNPs on the surfaces were 643.98 ± 26.31, 641.17 ± 95.27, 662.47 ± 12.20, and 648.94 ± 36.30 pieces/μm^2^. Statistical analysis showed no significant difference among this group of all types of D-AuNPs at the 0.05 significance level indicating D-AuNPs coating completed within 7.5 min.

**Figure 4:**
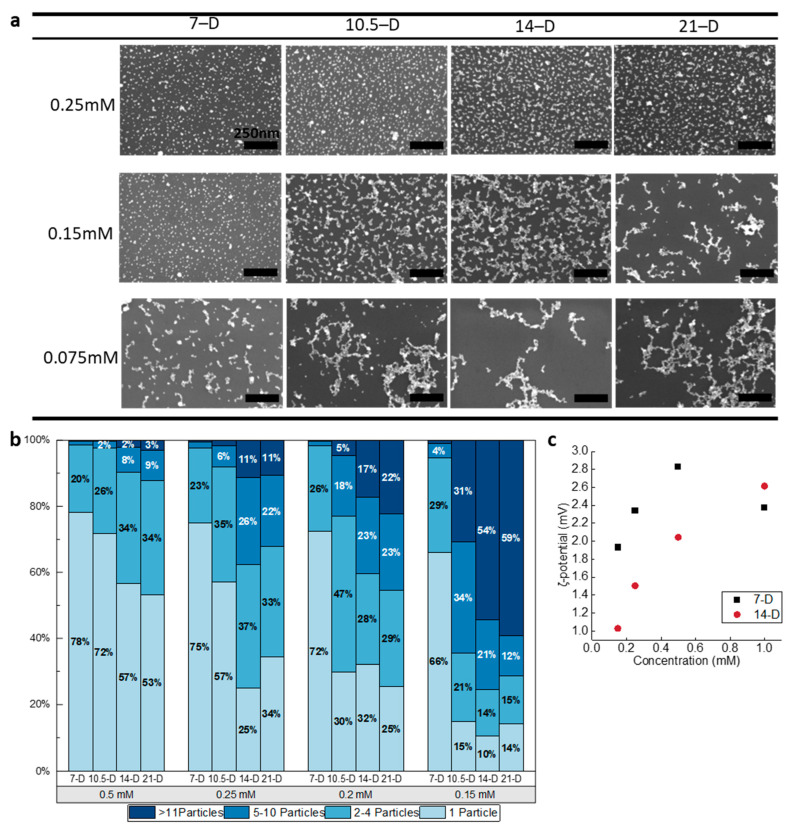
Patterned D-AuNP coating via dopamine amount and particle concentration manipulation. (**a**) D-AuNP coating on a silicon wafer using different particle concentrations. Top: 0.25 mM; middle: 0.15 mM; bottom: 0.075 mM. Left to right: 7–D, 10.5–D, 14–D, 21–D AuNPs. All coated overnight (scale bar: 250 nm). (**b**) Percentage of single particles, 2–4 particles clusters, 5–10 particles clusters, and more than 11 particles clusters counted from the SEM images above. (**c**) Zeta potential of D-AuNPs in 70% ethanol/water at different concentrations from 0.15 mM to 1 mM.

Interestingly, the D-AuNPs of varied dopamine densities displayed distinct coating patterns at lower concentrations, such as 0.15 mM (Figure 4a). For instance, less dopamine-modified AuNPs such as 7–D tended to form single particles at this concentration, while more dopamine-modified AuNPs such as 21–D severely aggregated on the surfaces. Additionally, a clear trend from 7–D to 21–D was observed, indicating that the coating pattern began with single-particle coating and gradually changed to a small aggregation (2–10 particles), finally reaching unevenly aggregated sedimentation. These findings suggest that D-AuNPs could be used as an effective coating material with a controllable coating pattern, which can be achieved by fine-tuning the dopamine amount and nanoparticle concentrations.

The patterned coating using D-AuNPs was achieved by controlling the extent of dopamine modification on the AuNP surfaces and the concentration of D-AuNPs in the coating systems (Scheme 1). D-AuNP coating patterns were categorized into four levels based on aggregation state: level 1 is dominated by single particles, level 2 is slightly aggregated but evenly coated particles (2–4 particles), level 3 is larger aggregated but evenly coated particles (5–10 particles), and level 4 is larger aggregated particles (>11 particles) beginning to coat unevenly. SEM images clearly show that the aggregation level increased as the concentration of D-AuNPs decreased or the dopamine amount increased (Figure 4a).

D-AuNPs with a higher dopamine density tended to form higher levels of aggregation at higher concentrations. From the result, 7–D AuNPs began to perform significant aggregation coatings when lower than 0.15 mM, while 14–D AuNPs required a concentration of 0.25 mM or higher to start forming similar coatings (Figure 4a). Single and cluster nanoparticles were counted via ImageJ, and the calculated distribution percentages are included in Figure 4b. At a 0.5 mM coating concentration, single particles and all D-AuNPs coated as single nanoparticles or clusters with less than four nanoparticles were predominant on D-AuNP-coated surfaces. For example, 44% of 7–D AuNP-coated surfaces were single particles and only 3% of clusters contained more than 10 particles (Figure 4b).

However, the percentage of single particles continuously decreased in all as the concentration of D-AuNPs decreased from 0.5 mM to 0.15 mM. This is especially true for 14–D and 21–D AuNPs, as single-particle percentiles on their coated surfaces dropped to 10% and 14%, respectively, at 0.15 mM. Under the same conditions, particle clusters containing more than 10 particles started to appear on the 7–D AuNP-coated surface at 2%. The number of clusters of more than 10 nanoparticles on 14–D and 21–D AuNP-coated surfaces reached 54% and 59%, respectively (Figure 4b). It is clear that the coating patterns changed gradually as the concentration of D-AuNPs decreased and the amount of dopamine increased. Increasing the amount of dopamine or decreasing the concentration of D-AuNPs resulted in elevated aggregation levels. Higher concentrations of particles may be needed if more single-particle coating is desired. To reveal the mechanisms behind the AuNP-concentration-affected surface coating, the zeta potential (ζ-potential) of D-AuNPs at different concentrations was assessed. The zeta potential was measured in 70% ethanol/water solution in agreement with the coating conditions. In addition, after being harvested from an acidic solution and resuspended in an ethanol-dominant solution, ions were mostly removed, thus, lowering the charged species in the solution. Given that the different viscosity and dielectric constant is different from water, the slipping plane charge would have smaller values. The zeta potential decreased as the concentration of D-AuNPs decreased. A single-particle coating could not be maintained once the zeta potential was lower than 1.5–2 mV. At 0.15 mM, 7–D AuNPs still had a higher zeta potential than 1.5 mV, while 14-D AuNPs reached 2 mV at 0.5 mM (Figure 4c). These results suggest that the coating pattern of D-AuNPs can be controlled by adjusting the concentration of D-AuNPs and the amount of modified dopamine; both factors can affect the pattern individually and synergistically. All changes in AuNP surfaces and concentrations eventually affected the zeta potential of the nanoparticles, leading to stability variations and these three patterns.

**Figure 5:**
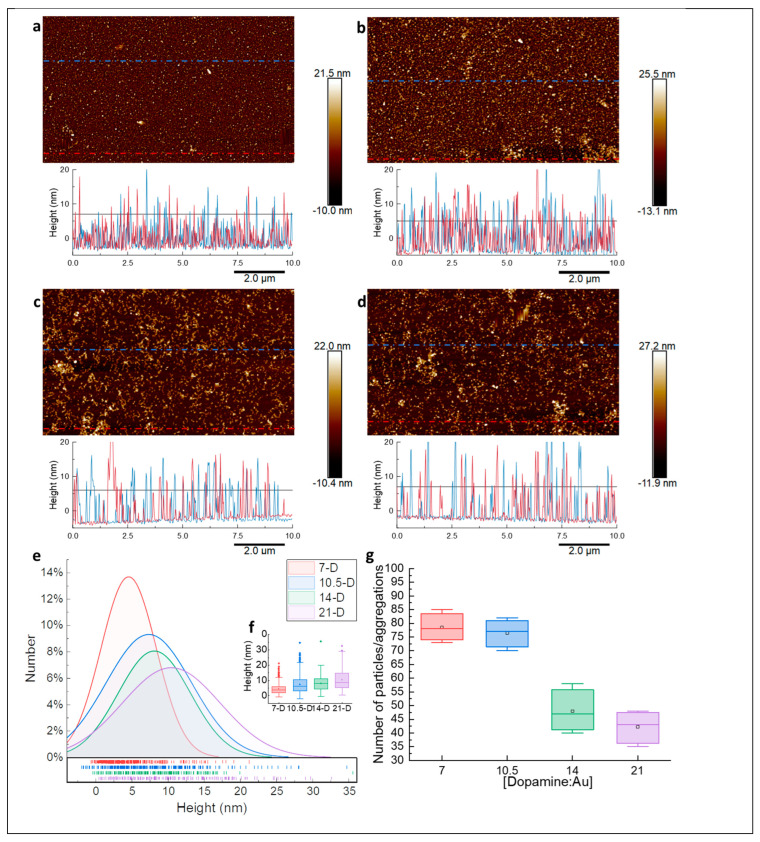
Surface morphology and particles/aggregations counting of patterned D-AuNP coating. (**a**–**d**)AFM images and related height measurement of 0.15 mM 7–D AuNPs (**a**), 10.5–D AuNPs (**b**), 14–D AuNPs (**c**), 21–D AuNPs (**d**); (**e**) height distribution measured from AFM; (**f**) actual height measured from AFM; (**g**) number of particles/aggregations per 10 μm range.

Furthermore, atomic force microscopy (AFM) was utilized to evaluate the height of D-AuNPs at 0.15 mM (Figure 5a–d). Despite the increased aggregations at this concentration, a few large clusters of more than 11 particles were observed. To investigate the actual height of each cluster, several lines were selected from each AFM image to perform the section measurement using the software NanoScope Analysis 2.0. The height of the blue and red lines (Figure 5a–d) represent a typical region plotted in the chart below each image. From these charts, most cluster heights are less than 10 nm above the baseline. Given the 8–10 nm physical diameters measured via SEM (Figure 2b), and most clusters remain in a single layer. From the red and blue lines, the number of peaks also decreased while the grafted dopamine density increased, which means that less clusters or nanoparticles were detected. This is because there are fewer single particles coated and some of them aggregate together to form small aggregations. The height distribution of all objects on each surface was calculated from several different section lines (Figure 5e). The height range increased from ~15 nm to ~35 nm as small aggregations became dominant. For 7–D AuNPs, most particles remained under 10 nm, suggesting a single-AuNP-particle layer coating. From 10.5–D to 21–D AuNPs, the mean and maximum height values started to increase, while the minimum value remained the same (Figure 5f). All D-AuNPs have a significantly different height distribution except for 10.5-D and 14-D, indicating different heights of small aggregation and single particles. However, the actual height difference was not comparable to the diameter of a AuNPs (8–10 nm). Such a height difference can be attributed to the squeeze or unbalanced aggregation rather than the formation of AuNP multilayers. That is, some nanoparticles within an aggregate may exhibit a slightly higher protrusion. In addition, 14–D and 21–D AuNP-coated surfaces have significantly less pieces than 7–D and 10.5–D AuNPs (Figure 5g). Given the aggregation level increase, the number of particles/aggregations decreased along with the dopamine amount changes. When higher dopamine amount or lower D-AuNP concentrations were used, the coating pattern tended to be aggregated and there was a lower coating density, and vice versa. Such equilibrium provided guidance for selecting the correct dopamine amount and D-AuNP concentration combination.

D-AuNPs demonstrated a rapid, uniform monolayer deposition, as well as patterned aggregation-mediated coating under certain conditions. D-AuNPs exhibit a net positive charge within a coating system, while a silicon wafer surface possesses a net negative surface charge around a neutral pH; D-AuNPs would be attracted to surfaces as they come into contact with them [46]. Once D-AuNPs attach to the silicon surface, a hydrogen bond between dopamine and silanol forms. Close contact ensures additional van der Waals interactions, which helps avoid removal. Such a combined attraction is strong enough against most rinses. Upon exposure to atmospheric oxygen, dopamine is oxidized and forms a stronger interaction with the surface. For lower particle concentrations, individual particles’ zeta potentials approach neutrality; thus, single particles have less attraction to the surface and less repulsion to one another. Therefore, they prefer to form small aggregations in solutions first, and then adhere to surfaces. Upon aggregation, they carry more charges and become heavier. Such clusters would need a longer time to gradually deposit on surface. However, D-AuNP deposition was limited to a monolayer in our study. At higher D-AuNP concentrations, the higher surface charge and high charge density provided a repulsive potential. However, these interactions only occurred over short distances. Based on the interparticle spacing visualized on the surface via SEM, double-layered repulsion is more reasonable. The dopamine layer is sufficient to form a sufficient hydration layer surrounding each particle, and more dopamine attracts more water molecules, as confirmed by the DLS results. Once a monolayer is formed, the surface charge and energy change to stop an additional layer, and aggregation occurs even within one monolayer. As the particle concentration decreased, electrostatic repulsion decreased and non-electrostatic contributions (e.g., hydrogen bonding) led to the formation of larger clusters. The coating pattern resembled the Volmer–Weber (VW)-like mode and particles formed small aggregations in a solution, and then were deposited on the surface.

## 4. Conclusions

Our research focused on novel adhesive AuNPs for controlled surface coatings. We could achieve a pattern-controllable coating by adjusting both the dopamine grafting density and adhesive AuNP concentrations guided by the equilibrium between aggregation level and cluster number. These adhesive AuNPs offer a simple and efficient way to modify surfaces without any pretreatment. The colloidal stability of adhesive AuNPs was ensured through protection with borate and can be easily activated by deprotection to achieve excellent coating surfaces. Our findings offer a promising avenue for developing new coating materials for a wide range of applications.

## Data Availability

Data are contained within the article.
